# Management of traumatic hemipelvectomy through ERAS pathway: A case report

**DOI:** 10.1097/MD.0000000000012251

**Published:** 2018-09-07

**Authors:** Nawin Ghimire, Zhao Kui Yan, Yue Fang, Walter Munesu Chirume, Yun Yang

**Affiliations:** Department of Orthopedics, Sichuan University, West China Hospital, Chengdu, China.

**Keywords:** amputation, enhanced recovery after surgery, hemipelvectomy, multidisciplinary coordination, multiple trauma

## Abstract

**Rationale:**

Traumatic hemipelvectomy is a rare but life-threatening injury that involves separation of the pelvic ring from pubic symphysis usually results from high energy trauma and associated with other injuries.

**Patient concern:**

In this report, we describe a case of traumatic hemipelvectomy, who presented in hemorrhagic shock associated with other injuries such as: right groin injury with limitation of passive movement of right hip and knee joint, left pelvic visceral protruded out, and wrapped by peritoneum, all of the vulva and anal tear, lumbar vertebrae transverse process fracture.

**Diagnoses:**

Traumatic hemipelvectomy.

**Interventions:**

The patient was managed through enhanced recovery after surgery (ERAS) pathway with multidisciplinary coordination.

**Outcomes:**

Patient was able to walk with prosthesis or crutch, with associated injuries managed appropriately. The course was complicated with hemorrhagic shock and infection which were dealt promptly, with good recovery.

**Lessons:**

In our case, the multimodal management through ERAS path has helped decrease stress level, decrease complication, decrease morbidity, decrease the length of stay in the hospital, and aid in faster recovery.

## 1 Introduction

Traumatic hemipelvectomy or hindquater^[1,2]^ amputation is rare but life-threatening injury^[3–12]^ that involves separation of the pelvic ring from sacroiliac joint and pubic symphysis. It results secondary to high velocity trauma.^[1,3,8,12,13]^ Associated injuries include gastrointestinal, urinary and less common as contralateral limb injury, peritoneal injury, intraabdominal injury, and perianal injury.^[4–8,10,13,14]^ Enhanced recovery after surgery (ERAS) refers to the rapid recovery of patient in the postoperative period by optimizing the effective treatment measures which is confirmed by a series of evidence-based medicine, to reduce the mental and physical stress response in the patient, reduce the complications, shorten the length of stay in hospital, reduce the risk of readmission, reduce morbidity, and mortality. Although ERAS was initially developed and intended for use in colorectal surgical pathways, its scope has widened in other surgical fields such as cardiothoracic surgery, gynecology surgery, urology surgery, orthopedic surgery, and other field, and has achieved good result.^[15]^ This is the first reported case managed with ERAS pathway with multidisciplinary coordination in traumatic hemipelvectomy. The ERAS pathway with multidisciplinary coordination in our case intended to address different element such as preoperative education, stress reduction, decrease complication, decrease cost, decrease the length of stay, and decrease morbidity.

## 2 Case report

**Figure 1:**
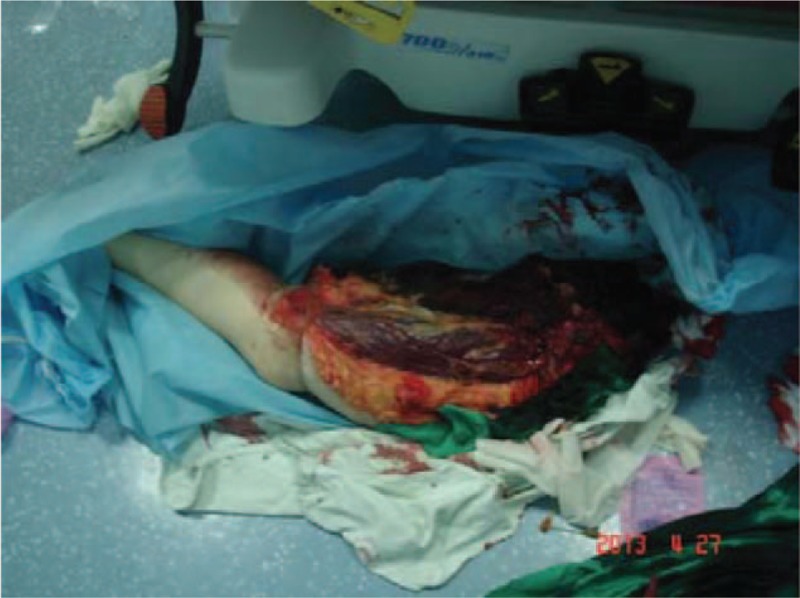
Photograph of posttraumatic nonviable left leg.

A 19-year-old, previously healthy, female was accidentally crushed by truck resulting in left hip hemipelvectomy, left hip stump bleeding on April 27, 2013. She was treated with compression bandage in the emergency department of the local hospital and transferred immediately to our hospital, it took postaccidental 3 hours to reach our hospital. Initial examination on arrival showed she was in shock with indifferent consciousness, her left leg was mangled and nonviable with left hip stump dressing. Immediate antishock treatment initiated with wound compression bandage, hemostasis was achieved, iv fluids and blood transfusion initiated, oxygen given, the patient was under continuous electrocardiography monitoring and other symptomatic treatment. She was resuscitated from shock and emergency consultation was done with gastrointestinal surgery, vascular surgery, burns and plastic surgery, and orthopedic surgery (Fig. 1).

**Figure 2:**
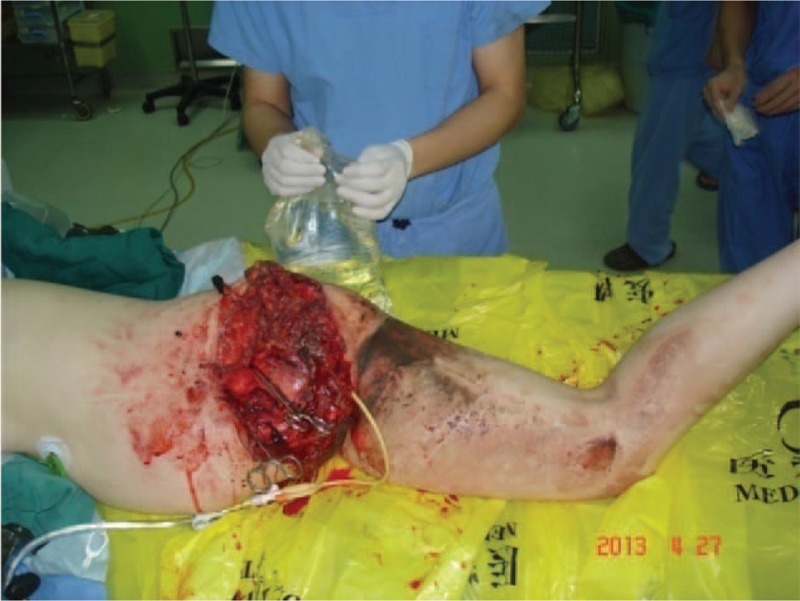
Photograph of posttraumatic amputated left pelvis prepared for irrigation of the wound.

Systemic examination was normal except her left lower abdominal wound margin extending to intestine and bladder. Her orthopedic injuries included amputated left pelvis, left lower pelvic organs were protruded out and wrapped by peritoneum, all of the vulva and anus were torn, the wound area was contaminated and actively bleeding, the right groin and perineal skin contusion with extensive skin abrasions, right knee and medial part of first great toe skin abrasion. The right hip joint, knee joint, and ankle joint with no obvious deformity but limited passive activity (Fig. 2).

**Figure 3:**
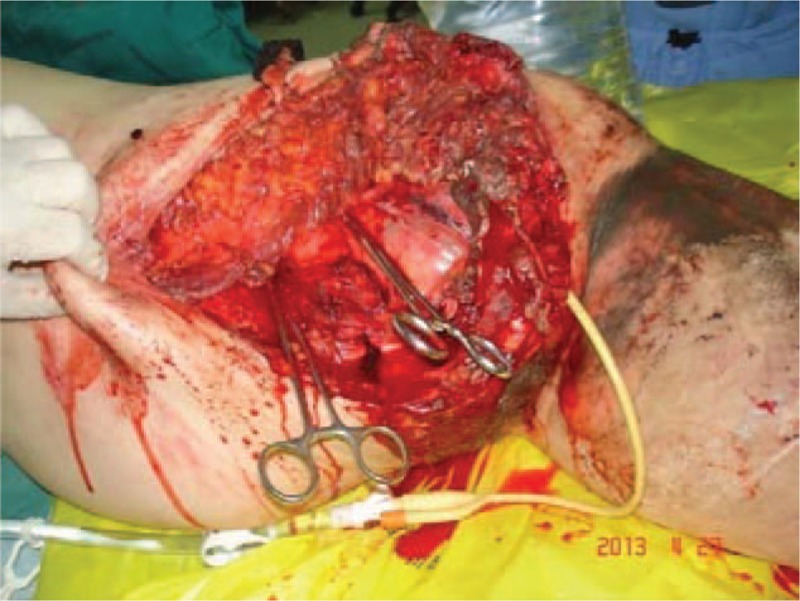
Initial photograph showing the extent of soft-tissue avulsion.

**Figure 4:**
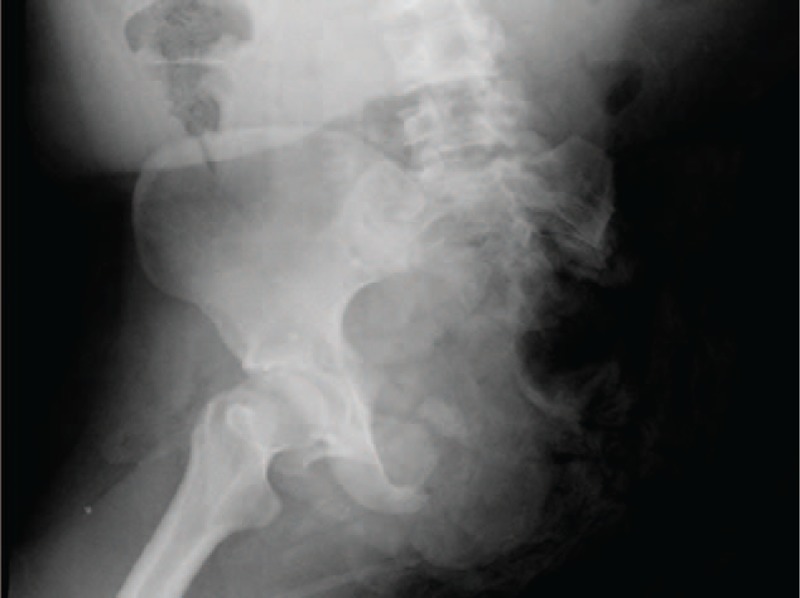
Initial radiograph after injury.

Emergency radiograph and abdominal CT showed left sciatic iliopectineal and left lower limb loss, rough cutting edge, local skin tear with multiple dense punctuate shadow. The left middle upper abdominal wall was swollen and accumulating. L3-5 left transverse process fracture, L5 vertebral right transverse process fracture, bilateral sacral wing fracture, right acetabulum fracture, comminuted fracture of a superior, and inferior branch of the right pubic ramus. There were contusion and laceration of the soft tissue of the pelvic floor, with the pelvic wall and the pelvic cavity scattered in the gas accumulation. Small compact shadow beside the caudal vertebra, ruled out the fracture of the caudal vertebra (Figs. 3 and 4).

**Figure 5:**
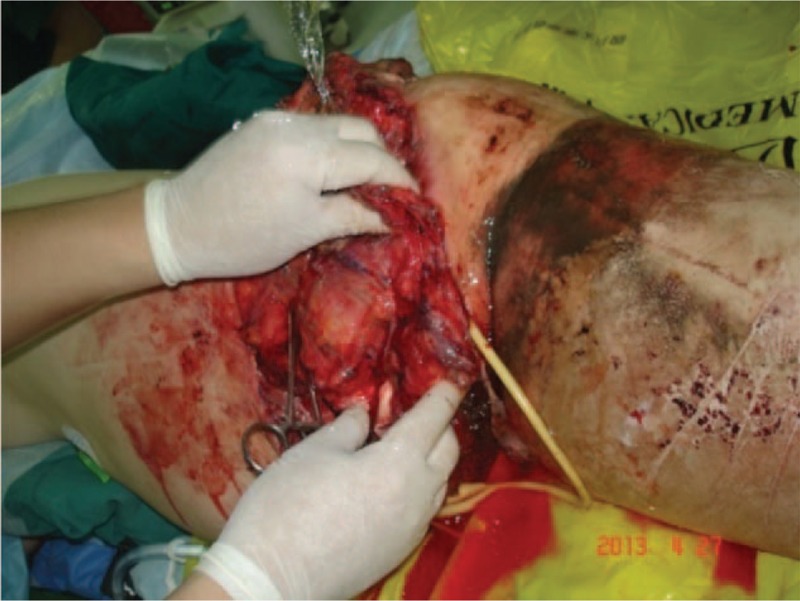
Intraoperative photograph of repair of the defect.

**Figure 6:**
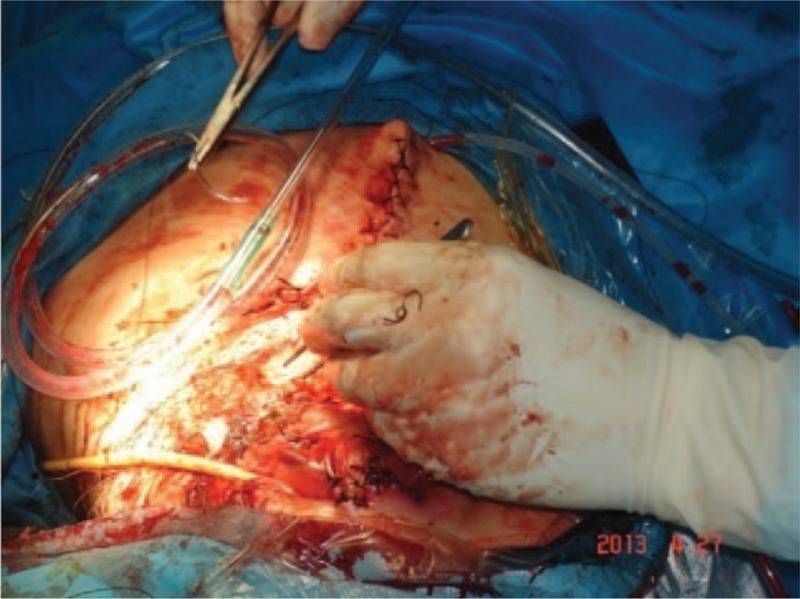
Photograph of skin closure of the amputated wound.

The patient and her family were explained about patient condition, the treatment modality, the possible complication, and encouraged for their active participation. After resuscitation from shock and emergency consultation with gastrointestinal surgery, vascular surgery, burns and plastic surgery, and department of orthopedics, considering her current critical condition, consent was taken for current treatment of stump wound debridement and suture drainage + laparotomy transverse colostomy, as a part of multistage surgical treatment for her condition. In operating theater, after successful general anesthesia, she was positioned in right lateral position. After drapping with aseptic technique, the wound was rinsed with hydrogen peroxide and saline to remove the contamination. After emergency ligation of the left internal and external iliac artery, excision of necrotic wound edge was done than intermittent suture for anal and perineal tear, and the continuous suture was applied for left abdominal wall large defect. There was minimal bleeding during this procedure. Then the patient was positioned supine, after confirming adequate anesthesia drapping was done with aseptic technique, the abdominal cavity was explored through a midline incision. Liver, spleen jejunum, ileum, and cecum were explored, that showed no obvious abdominal injury. The transverse colostomy was done and the abdominal cavity was closed. There was also minimal bleeding during this procedure. She received an intraoperative and postoperative blood transfusion. Considering her critical condition she was transferred to intensive care unit (ICU) for postoperative care and ventilator support. She was taken over by physician and team for ICU management (Figs. 5 and 6).

Department of Orthopedics, gastrointestinal surgery, burns and plastic surgery, urosurgery, obstetrics, and gynecology were involved for proper monitoring of wound dressing, colostomy care, prevention of complication, and to strengthen patient communication. Parental nutrition was started with adequate electrolyte supplement. She developed a fever on the first postoperative day than bedside chest X-ray was taken that showed normal pleural condition, blood coagulation report and lactic acid status evaluated were normal, and the specialist was consulted to evaluate wound and stoma. Gentamycin, cefuroxime, and metronidazole were discontinued and piperacillin-tazobactam combined with the hepatoprotective drug was initiated. Fever subsided in days, nutritional support was started and increased gradually in days from parental than small oral glucose + small calorie parental than the addition of fat emulsion as tolerated. She was weaned from the ventilator and finally extubated. She was transferred to the orthopedic ward for further management. Excision of necrotic tissue and dressing were continued and when wound showed some granulation, it was assisted by burn and plastic surgery team. Meanwhile, the patient developed abdominal pain resolved by temporary fasting, antacid, and rehydration and ontrast enhanced computed tomography scan taken after gastrointestinal surgery consultation was normal. On the postoperative 22nd day, she developed intermittent fever subsided with antipyretic and oral cotrimoxazole, wound examination showed minimal bleeding and more exudates. Blood examination showed increase in alanine transaminase (178 IU/L), aspartate transaminase (126 IU/L), decrease albumin (27.9 g/L), total protein (47.3 g/L), hemoglobin (HB; 105 g/L), and hematocrit (0.23 L/L) with other in normal range. Now the patient was coordinated with infectious disease department, hematology department, and burn and plastic surgery department. Piperacillin-tazobactam was discontinued, and cefoperazone-sulbactam was enabled with a hepatoprotective drug to reduce glutathione and electrolyte correction continued.

Next day, blood transfusion was planned as patient appeared pale but the patient was transferred to ICU after worsening of condition: decrease blood pressure, increase pulse: 123 bpm, respiratory rate: 22 to 43/min, the wound dressing of left perineum were percolated. Emergency blood gas analysis showed: HB 4.0 g/L, partial pressure of oxygen 54 mm Hg, sodium 132.7 mmol/L, chlorine 113.3 mmol/L, lactate 4mmol/L, whole blood base 3.0 mmol/L. With positive transfusion therapy and noninvasive ventilator-assisted ventilation, antibiotic, electrolyte correction, nutritional support, rehydration, daily dressing, and other symptomatic and supportive treatment, the patient condition was stabilized and transferred back to the orthopedic department. She complained of phantom limb pain which was managed with gabapentin and methylcobalamin. The regular dressing was continued and when wound appeared fresh burn and plastic surgery department were consulted.

**Figure 7:**
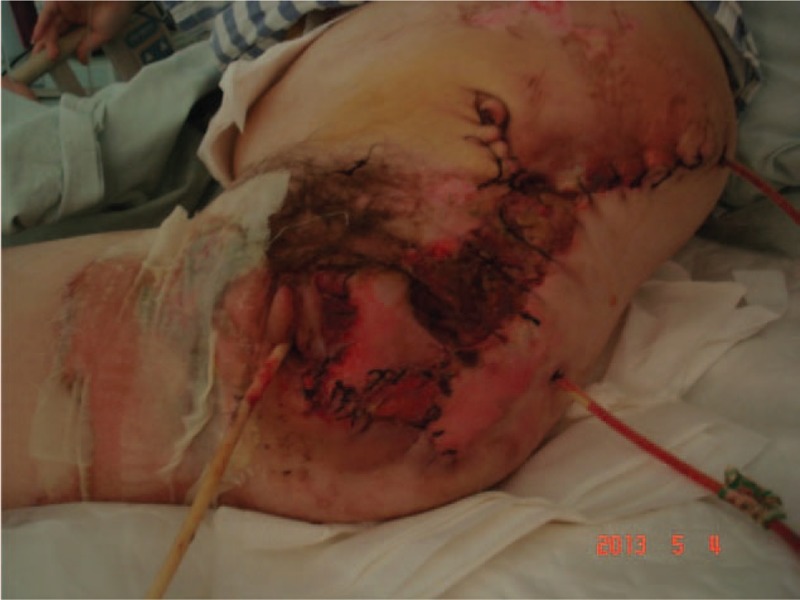
Photograph of right anterior thigh scab wound after free graft taken.

**Figure 8:**
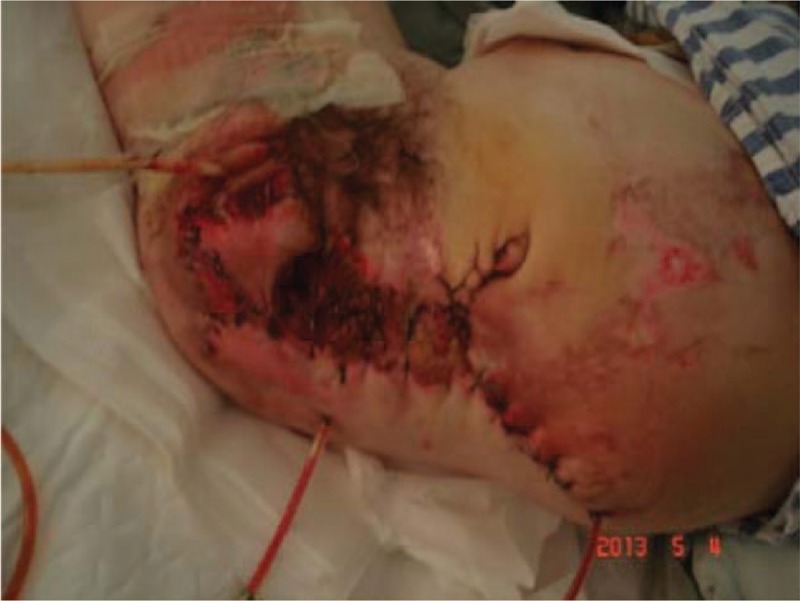
Photograph after skin graft repair for left stump defect.

**Figure 9:**
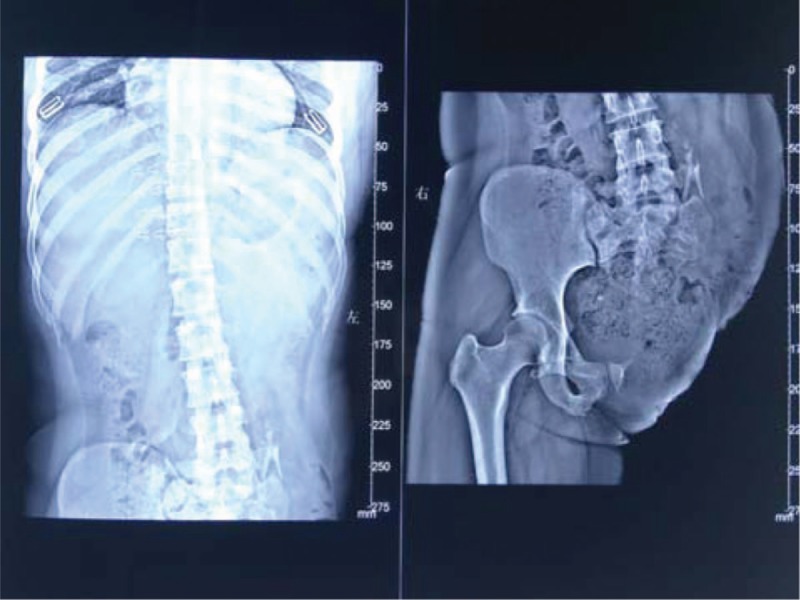
Recent computed tomography scan about 5 years later showing scoliosis of lumbar vertebrae.

In the postoperative 39th day after abundant granulation tissue was seen, the case was discussed with the department of burn and plastic surgery and a skin graft was planned. Next day, free skin graft transferred to left lower abdomen from right anterior thigh (Figs. 7–9). Examination showed the left lower abdominal wound as 20 cm × 10 cm and right groin had 6.0 cm × 4.0 cm granulation wound that was healthy with minimal secretion. There was perineal secretion for few days which was dry after perineal hygiene. Eighteen days later, the patient was transferred to physiotherapy and rehabilitation department. Therapy was initiated to improve the strength of right lower extremity and improve the ability of adductor longus, enhanced upper limb muscle strength training. Tramadol and Rui Calgary music therapy were given for phantom limb pain.

With 6 months of interval rehabilitation therapy patient daily life ability score improved from 35 to 96 out of 100, examination showed the amputation site wound of 15 cm × 10 cm with little yellowish graft exudates, on the lateral side of the right thigh, there was about 10 cm × 10 cm of scab wound with limited function of right hip and knee joint. Right groin had 20 cm × 5 cm abrasion which was dry and healthy, there was artificial stoma in the abdomen with colostomy margin red and humid, no break and covered with pocket, with surrounding skin clean and dry. The patient complains of occasional phantom limb pain significantly affecting sleep which was treated symptomatically and with methylcobalamin, occupational therapy, and rehabilitation training. She could wear prosthetic limbs and walk with the aid of walking aids. Her colostomy was anastomosed with anus a few months later by the department of gastrointestinal surgery.

Recently she is unemployed, unmarried stays with her parents who take care of her. She complains of phantom limb pain not responsive to analgesic, so she does not use it. She has a prosthetic leg but does not prefer it as it is inconvenient to walk with it. She usually walks with single crutch up to 100 m.

## 3 Discussion

The ERAS does not have any solitary definition, all of them describing characteristic pathway as accelerated rehabilitation or fast-track surgery.^[15]^ ERAS refers to the rapid recovery of patient in the postoperative period by optimizing the effective treatment measures which is confirmed by a series of evidence-based medicine, to reduce the mental and physical stress response in the patient, reduce the complications, shorten the length of stay in hospital, reduce the risk of readmission, reduce morbidity, and mortality. Although ERAS was initially developed and intended for use in colorectal surgical pathways, its scope has widened in other surgical fields such as cardiothoracic surgery, gynecology surgery, urology surgery, orthopedic surgery, and other field, and have achieved good result.^[15]^ The general pathway is a dynamic and fluid pathway, and the concept of ERAS is still in process of development with great variability around the world.^[16]^ In our case, the ERAS pathway with multidisciplinary coordination was intended to address different element such as preoperative education, stress reduction, decrease complication, decrease cost, decrease the length of stay, and decrease morbidity.

Traumatic hemipelvectomy is rare but life-threatening injury results from high-velocity trauma and usually associated with other injuries such as gastrointestinal, urinary, contralateral limb, peritoneal, intraabdominal, and perianal injury. Treatment of hemipelvectomy requires early resuscitation and early involvement of multiple departments including orthopedic, general surgery, urosurgery, burn and plastic surgery, psychiatry, physiotherapy, and rehabilitation.^[14]^ Teams taking over the patients do not have sufficient idea whether the patient they are receiving be presenting differently and potentially in a better shape because of lack of insight into the previous care process, so to overcome this serious issue ERAS pathway has to be built with everyone involved in the chain of patient journey including surgeons, anesthetists, dietitians, physical therapists, and nurses.^[17]^

In our case, care was given for proper coordination between multiple departments so that we could enhance the care of the patient. Coordination between the orthopedic surgery and gastrointestinal surgery department addressing the most critical condition initially was beneficial to the patient, as the life-saving procedure by obtaining the prompt hemostasis with ligation of vessels. Debridement of necrotic tissue and transverse colostomy were equally beneficial as they eliminate the focus of sepsis by removing the source of contamination, removing devitalized tissue, and maintaining continence. Later on, the colon was anastomosed with anus when the perianal wound was healthy, reducing the morbidity. Coordination between surgery department, orthopedic department, internal medicine department, gynecology department, burn and plastic surgery department, infectious disease department, dietitian, and nursing department were crucial for proper wound care, prompt management of complications, decrease metabolic stress, leading to decrease in the length of stay, and decrease the cost of management. The decision to take anterior thigh graft with coordination with burn and plastic surgery after the appearance of granulation tissue was also an important part of enhanced recovery pathway. The graft taken from anterior right thigh was successful graft, with no complication. This was helpful to address every element of ERAS. Physiotherapy and occupational therapy, which played important role in enhancing recovery path by decreasing length of stay, lead to decrease in the cost.

Preoperative education is believed by informing the patient's expectations about each and every aspect of their journey facilitate their smooth recovery by decreasing their anxiety level knowledge and ability to perform exercise postoperatively.^[16]^ In our case, preoperative information to the patient and her family lead to decrease in the anxiety level, increase the psychologic acceptance, better care, early mobilization, and self-participation aiding in faster recovery. Furthermore, the caretaker counseled and trained earlier aided the recovery by appropriate management of patient at the hospital and home.

Another key factor for enhancing recovery is a reduction in stress. The components of stress such as anxiety, pain, tissue damage, hemodynamic disturbance, hypoxia, and sleep disturbance were dealt promptly. Care was given that metabolic stress due to electrolyte imbalance, nutritional deficiency, fluid deficiency, pain, fluid maintenance, and oxygen inadequacy could be promptly and adequately addressed. Considering the patient physiologic state multistaged surgery was performed.

## 4 Conclusion

Management of a patient with ERAS pathway including multidisciplinary coordination in traumatic hemipelvectomy is an ongoing matter of study, we have experience that multimodal management with multidisciplinary coordination had lead to decrease stress level, decrease complication, decrease morbidity, decrease the length of stay in the hospital, and aid in faster recovery.

## Author contributions

**Data curation:** Zhao K Yan.

**Supervision:** Yue Fang.

**Writing – original draft:** Nawin Ghimire.

**Writing – review & editing:** Nawin Ghimire, Zhao K Yan, Walter M Chirume, Yun Yang.
